# Toxic Peptides Occur Frequently in Pergid and Argid Sawfly Larvae

**DOI:** 10.1371/journal.pone.0105301

**Published:** 2014-08-14

**Authors:** Jean-Luc Boevé, Raoul Rozenberg, Akihiko Shinohara, Stefan Schmidt

**Affiliations:** 1 Royal Belgian Institute of Natural Sciences, Bruxelles, Belgium; 2 Institute of Condensed Matter and Nanosciences, Université Catholique de Louvain, Louvain-la-Neuve, Belgium; 3 Department of Zoology, National Museum of Nature and Science, Tsukuba-shi, Ibaraki, Japan; 4 Zoologische Staatssammlung, Staatliche Naturwissenschaftliche Sammlungen Bayerns, München, Germany; University of South Florida College of Medicine, United States of America

## Abstract

Toxic peptides containing D-amino acids are reported from the larvae of sawfly species. The compounds are suspected to constitute environmental contaminants, as they have killed livestock grazing in areas with congregations of such larvae, and related larval extracts are deleterious to ants. Previously, two octapeptides (both called lophyrotomin) and three heptapeptides (pergidin, 4-valinepergidin and dephosphorylated pergidin) were identified from three species in the family Pergidae and one in Argidae. Here, the hypothesis of widespread occurrence of these peptides among sawflies was tested by LC-MS analyses of single larvae from eight pergid and 28 argid species, plus nine outgroup species. At least two of the five peptides were detected in most sawfly species, whereas none in any outgroup taxon. Wherever peptides were detected, they were present in each examined specimen of the respective species. Some species show high peptide concentrations, reaching up to 0.6% fresh weight of 4-valinepergidin (1.75 mg/larva) in the pergid *Pterygophorus* nr *turneri*. All analyzed pergids in the subfamily Pterygophorinae contained pergidin and 4-valinepergidin, all argids in Arginae contained pergidin and one of the two lophyrotomins, whereas none of the peptides was detected in any Perginae pergid or Sterictiphorinae argid (except in *Schizocerella pilicornis*, which contained pergidin). Three of the four sawfly species that were previously known to contain toxins were reanalyzed here, resulting in several, often strong, quantitative and qualitative differences in the chemical profiles. The most probable ecological role of the peptides is defense against natural enemies; the poisoning of livestock is an epiphenomenon.

## Introduction

Toxic peptides are parts of the tremendous wealth of bioactive metabolites in microbes, plants and animals. They are known from bacteria, soil fungi, mushrooms, (some) plants, sea anemones, cone snails, scorpions, spiders, bees, wasps, frogs, and snakes [1]–[6]. Some of these toxic peptides are quite unique in containing D-amino acids [2], although both toxic and non-toxic D-amino acids and D-amino acid proteins have been detected in prokaryotes and most eukaryotes, except plants [7], [8]. Another rare feature of peptides from natural sources is their inclusion of phosphoserine, an example being the pentapeptide alphostatin isolated from a strain of the bacterium *Bacillus megaterium* [9]. Only one source in nature is known in which the peptides combine a high proportion of D to L amino acids with the presence of phosphoserine; these peptides were discovered in sawflies (Insecta, Hymenoptera).

Since the mid-20^th^ century there have been uncommon but repeated reports of livestock dying after ingesting certain insects. Hundreds of cattle, sheep, goats, and pigs have been found dead, with significant economic consequences to the farmers [10]–[12], first in eastern Australia [13], [14], then in Denmark [15], and South America [12], [16], [17]. The mammals perished after grazing in areas showing congregations or outright outbreaks of larvae belonging to one of two sawfly families, Pergidae or Argidae. Autopsies of carcasses revealed liver necroses and stomachs filled with the larvae. On occasions in Australia and Uruguay a kind of addictive behavior was observed, with cattle fighting each other for the opportunity to ingest more larvae once they had tasted them for the first time [14]. This behavior, while impossible to interpret physiologically at this time, appears to explain why mass quantities of insects were ingested.

On another level, the toxic peptides can affect biological control programs. The Australian sawfly species *Lophyrotoma zonalis* (Pergidae) is a potential control agent of the paperbark tree, *Melaleuca quinquenervia* (Myrtaceae), an invasive plant in Florida, but the sawfly has not been introduced there as the risks of environmental contamination by the toxins were considered as too high [18]–[21]. Similarly, the pergid *Heteroperreyia hubrichi* was initially selected as a candidate for biological control of the Brazilian peppertree, *Schinus terebinthifolius* (Anacardiaceae), an invader to Florida, California and Hawaii. However, the introduction of that sawfly has been delayed, again because of its potential for poisoning native wildlife and domesticated animals that may consume the insect larvae [22].

The ecological implications of the toxins at the two levels, of killing livestock and potentially contaminating the environment, prompted us to investigate the occurrence of the toxins across a broader range of sawfly species. The first identified oligopeptide was discovered in the pergid *Lophyrotoma interrupta*, thus called lophyrotomin (LGln); it is an octapeptide with four D-amino acids [23]–[25]. Subsequently, a closely related octapeptide (LGlu) and three heptapeptides were discovered in other sawfly species ([26], [27]; Table 1): pergidin (Perg), 4-valinepergidin (VPerg), and dephosphorylated pergidin (dpPerg). The heptapeptides contain five D-amino acids, and Perg and VPerg also contain phosphoserine. The compounds are highly toxic to vertebrates, as demonstrated by the following examples of lethal dose (LD) values: LD_50_ ≈2 mg LGln/kg mice (intra-peritoneal injection); LD_50_ ≈10 mg Perg/kg mice (intra-peritoneal injection); LD = 6 g fresh weight (FW) *L. interrupta* larvae/kg sheep (oral dosing); medium LD = 3–9 g dry weight (DW) *L. interrupta* larvae/kg chicken (oral dosing); and lethal single doses of 10–40 g FW *Perreyia flavipes* larvae/kg sheep within 68–14 hrs [11], [12], [23], [24], [28]–[30].

**Table 1:** Molecular weights and structural composition of the five toxic peptides known from larvae of Pergidae and Argidae.

Peptide name; abbreviation^a^	MW	Structure
Pergidin; Perg	864	(L)pGlu-(D)Ala-(D)Val-(L)Leu-(D)Val-(D)Ser(PO_3_H_2_)-(D)Trp(OH)
4-Valinepergidin;VPerg	850	(L)pGlu-(D)Ala-(D)Val-(L)Val-(D)Val-(D)Ser(PO_3_H_2_)-(D)Trp(OH)
Dephosphorylatedpergidin; dpPerg	784	(L)pGlu-(D)Ala-(D)Val-(L)Leu-(D)Val-(D)Ser-(D)Trp(OH)
Lophyrotomin;LGln	1039	C_6_H_5_CO-(D)Ala-(D)Phe-(L)Val-(L)Ile-(D)Asp-(L)Asp-(D)Glu-(L)Gln
‘Lophyrotomin’;LGlu	1040	C_6_H_5_CO-(D)Ala-(D)Phe-(L)Val-(L)Ile-(D)Asp-(L)Asp-(D)Glu-(L)Glu(OH)

a Abbreviation used in the present study.

Molecular weights and structures of Perg, VPerg and dpPerg after MacLeod *et al.* [26], of LGln and LGlu after Oelrichs *et al.* [27].

Previously, the peptides had been detected and quantified in only four sawfly species: *L. interrupta*, *L. zonalis*, *P. flavipes* (Pergidae), and *Arge pullata* (Argidae) [24], [26], [27], [31]. Each of these species can occur in masses in the field, which facilitated the detection of the toxins. However, we started our study from the hypothesis that the toxins would be found also in related but non-pullulating sawfly species. Experiments with extracts from larvae of *Arge pagana*, a species common on roses in Europe, and from *A. pullata*, that were tested on ants (*Myrmica rubra* L., Formicidae) had caused the latter to show paralyzing effects [32]. These bioassay results indicated that *A. pagana* contains the toxic peptides just like *A. pullata*.

In the past, large batches of probably thousands of larvae were required to isolate, identify, and quantify the peptides, by using oven or freeze dried larvae. For the current study, we designed an extraction procedure using single larvae, and performed liquid chromatography–mass spectrometry (LC-MS) analyses. This allowed us for the first time, to include relatively rare target species and to estimate inter-individual variation in peptide profiles. The screening of numerous Pergidae and Argidae species reveals that most of them contain the peptides.

## Materials and Methods

### Taxon Sampling

Sawfly specimens were collected in the field, mainly in Australia, Europe and Japan, the taxon sampling comprising eight Pergidae and 28 Argidae species (Appendix S1). No specific permissions were required for collecting the insects, and no endangered or protected species were involved in these collections. Voucher specimens are deposited at the Royal Belgian Institute of Natural Sciences. As the specimens used in the chemical analyses had to be destroyed completely in the process, one or more specimens from the same population, if not from the same egg-batch, constitute the respective morphological voucher.

### Extraction of Larvae

**Figure 1:**
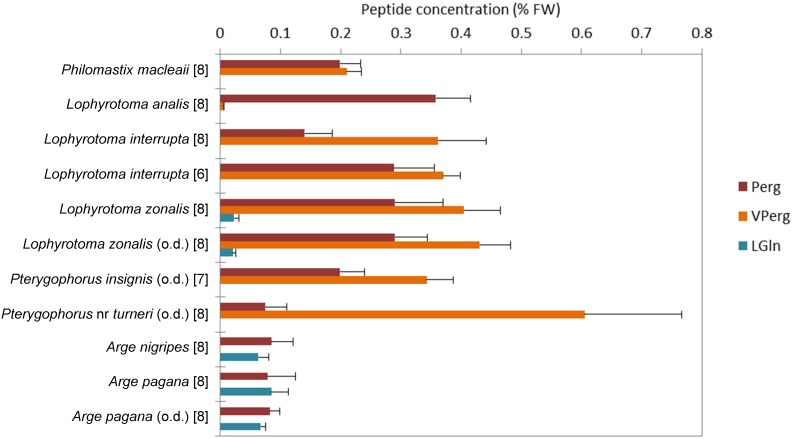
Concentration of the major peptides detected in six species of Pergidae and two species of Argidae. Peptide names abbreviated as in Table 1. Values expressed as means ± SD. Numbers of analyzed specimens given in square brackets. Prior to extraction and analysis, specimens were either kept in ethanol, or oven dried (o.d.).

**Figure 2:**
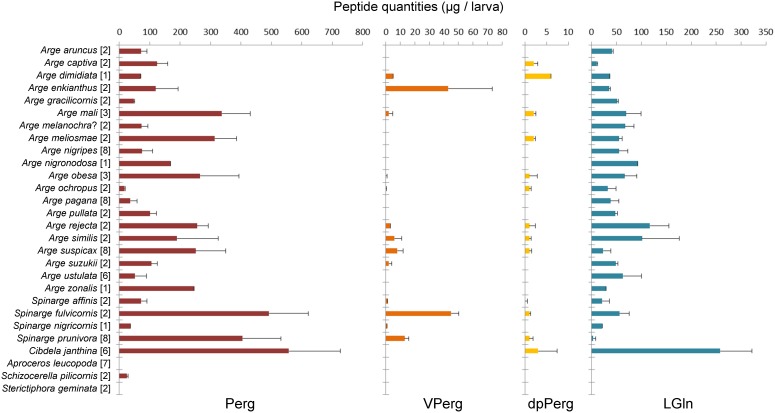
Absolute quantities of four peptides detected in 28 species of Argidae. Peptide names abbreviated as in Table 1. Values expressed as means ± SD. Numbers of analyzed specimens given in square brackets. Data for *Arge nigripes* and *Arge pagana* are those from Fig. 1.

For each of six pergid and two argid species, 6–8 specimens of full-grown or almost full-grown larvae were placed individually in a 1.5 ml microtube, weighed and then euthanized (see Fig. 1), either by adding 0.5–1.0 ml ethanol or by drying in an oven at 80°C for 20 hrs. Dried specimens were weighed again after the drying procedure. For all other species, 1–8 specimens per species were killed in ethanol (see Fig. 2). Generally these larvae were not weighed, but the approximate fresh weight (FW) was estimated from the body size (for the extraction and dilution procedure; see below). All specimens were stored at –30°C until extraction.

After numerous trials with various extraction procedures coupled with chemical analyses, the following protocol proved to give efficient, reproducible results on the content of toxic peptides in a single larva. The ethanolic extract (of non-dried specimens) was transferred to a 12 ml tube. The larva was thoroughly crushed in 1 ml of an equal volume ethanol–water solution (which also served for extractions from oven dried specimens). Then, the specimen was vortexed, and sonicated for 15 min at 50±5°C, followed by 5 min centrifugation at 8,000 rpm (Denver Instruments Microcentrifuge Force 7). The supernatant (extract n°1) was added to the tube, while 1 ml of 50% ethanol was added to the pellet remaining in the microtube. This sample was again vortexed, sonicated for 5 min, and centrifuged under the conditions stated above, which led to an extract n°2. Two to five extractions (with 5 min sonication) were performed, and the supernatants accumulated in the tube. The number of extractions depended on the FW of each larva, as follows: 2 (for determined or estimated larval weights <75 mg), 3 (76 to 150 mg), 4 (151 to 300 mg), or 5 (>300 mg). Ethanol (50%) was then added to these pooled extracts to obtain 3, 4, 5, and 6 ml of solution, respectively. The pooled extracts were stored at –80°C until dilution. Pilot trials showed that the extracts were generally too concentrated to allow peptide quantification by mass spectrometry. Using the aforementioned larval weight categories, three aliquots each of the pooled extracts were diluted to 1∶20, 1∶30, 1∶40, or 1∶50, respectively, prior to chemical analysis.

### LC-MS Analyses

We used an LC system from Thermo Separation Products (TSP, San Jose, California) that is equipped with a P1000XR pump and a TSP AS 3000 autosampler. Separation of peptides was performed on a C18 Ultrasep ES column (150×2.0 mm i.d., 7 µm; Sepserv GmbH, Berlin, Germany) using a linear gradient from 90% H_2_O (with 1% CH_3_CN and 0.1% HCOOH)/10% CH_3_CN to 10% H_2_O in 23 min. The flow rate was 0.2 ml min^−1^; the column was maintained at 30°C and the autosampler at 10°C. Mass spectra were acquired with a Quantum mass spectrometer (Finnigan MAT, San Jose, CA, USA) equipped with an electrospray ionization (ESI) source in the positive mode. ESI inlet conditions were: capillary heated at 380°C, sheath gas at 47 PSI, and auxiliary gas at 20 PSI. Collision induced dissociation was recorded at a relative collision energy of 30%.

### Peptide Quantification, Analytical Quality Controls, and Stability of the Peptides

The five peptides known from the literature to occur in larvae of Pergidae and Argidae (Table 1) were synthesized by, and purchased from, Biosyntan GmbH (Berlin, Germany) at >95% purity. They were analyzed by full MS^2^ on the [M+H]^+^ ions (Appendix S2). To differentiate between ions C^13^ of LGln (*m/z* = 1040) and C^12^ of LGlu (*m/z* = 1040), the latter peptide was analyzed using the single ion monitoring mode for the ion fragment *m/z* = 866, which caused no interference between the two ions.

Calibration curves for the five standard (*i.e.* synthesized) peptides were constructed over five concentrations in the range of 1–2000 ng/ml, and proved to be linear, with r^2^ values >0.99. The limit of detection for the peptides analyzed by LC–MS was in the 0.1 ng range, their limit of quantification being in the 1 ng/ml range.

The peptide concentrations in the samples were determined by comparing their ratios of peak areas to calibration curves. These concentrations (in ng/ml) were multiplied by one of the factors 60, 120, 200 or 300, depending on the two dilutions operated (*e.g.*, 3 ml of pooled extract diluted to 1∶20 leads to factor 60). The final concentrations (in µg/individual) were averaged over the three replicates (aliquots). For eight species where the precise FW of a larva was known (see Fig. 1), peptide concentration was expressed in % FW.

The recovery of the five peptides was determined using larvae of *Rhadinoceraea micans* and *Nematus miliaris* (Tenthredinidae), two species that had shown no detectable amounts of the peptides in previous LC-MS analyses. Specimens were extracted by the same procedure as all other specimens, except that after removal of ethanol 100% they were crushed not in ethanol 50% but in ethanol 50% plus 200 or 1000 ng/ml of the standards. Since these recovery experiments were carried out with larvae having a FW in the 76–150 mg range, the purpose was to obtain 24 and 120 µg/individual, respectively. The recoveries averaged over the five peptides actually yielded 24.5 and 125.4 µg/individual, with a coefficient of variation of 14% and 11%, respectively.

Stability of the peptides was monitored for 4.5 months in eight larvae of *L. zonalis*, using one of the three replicates available (and kept in ethanol 50%). They were stored at –30°C between the successive LC-MS analyses, which were performed at least once per month.

## Results

Toxic peptides occurred in each of the 6–8 individual larvae (with determined weight) from each of the six pergid and two argid species, at least by the constant presence of Perg (Fig. 1, Appendix S3). This was the major peptide in *L. analis*, whereas VPerg was the major one in the other pergid species. In contrast, LGln and especially Perg were major compounds in the Argidae. Our results invariably show that at least one peptide was present in each specimen of all these species. However, peptide quantities can vary among populations of the same species. Larvae from two populations of *L. interrupta* contained similar amounts of VPerg but various amounts of Perg, whereas two populations of *A. pagana* showed similar amounts of both Perg and LGln. The total amount of peptides ranged from ca. 0.4% to 0.8% FW in the Pergidae (Fig. 1). In *Lophyrotoma* spp. and *Pterygophorus* spp. only small amounts of dpPerg (<0.005% FW) were detected, and in some of the individuals only; LGlu was detected in only one of the two populations of *L. zonalis*, at 0.002% FW (Appendixes S1 and S3).

Perg and LGln were always detected in *Arge* (20 screened species) and *Spinarge* (4), and both peptides were detected in *Cibdela janthina*, whereas only Perg was present in *Schizocerella pilicornis* (Fig. 2). Among the Argidae, *C. janthina* reached the highest concentrations, with over 500 µg Perg and ca. 250 µg LGln per individual. The peptides VPerg and dpPerg occurred sporadically among argid species, and at relatively low mean concentrations never exceeding 60 and 7 µg/individual, respectively (Appendix S3).

No peptides were detected in any individuals of *Perga affinis*, *Pergagrapta polita*, *Aproceros leucopoda*, *Sterictiphora geminata*, nor in any of the outgroup taxa, which included sawfly and non-sawfly species (see Appendixes S1 and S3).

**Figure 3:**
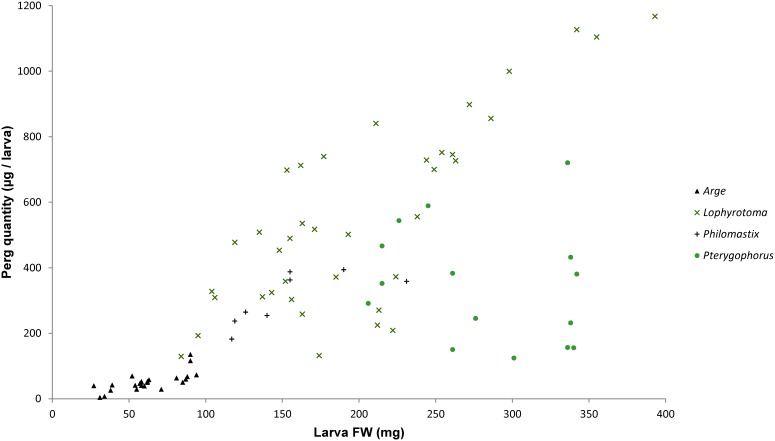
Fresh body weight and detected quantity of pergidin in individual larvae of species of Pergidae and Argidae. Species data from Fig. 1 combined at genus level, where applicable, *i.e. Arge nigripes* + *Arge pagana*; *Lophyrotoma analis* + *Lophyrotoma interrupta* + *Lophyrotoma zonalis*; *Philomastix macclaei*; *Pterygophorus insignis* + *Pterygophorus* nr *turneri*.

Body size is generally larger in the studied Pergidae than in the Argidae, but these differences were only partially reflected in the Perg contents (Fig. 3). Body weight correlated with the absolute quantity of Perg regardless of the taxa (shown in Fig. 1), except for the two *Pterygophorus* species that showed a proportionally low Perg concentration (Fig. 3). Concerning the methods of killing and storing the insects, *i.e.* in ethanol *versus* by drying, no significant differences between peptide profiles were noticed (Fig. 1), and a roughly constant DW/FW ratio of 20–25% was obtained for larvae of different species (value of FW and DW in mg as mean ± SD): *L. zonalis* (220±45 and 56±14; n = 8), *Pterygophorus insignis* (262±54 and 51±8; n = 7), *Pterygophorus* nr *turneri* (300±49 and 59±9; n = 8), *A. pagana* (56±7 and 12±1; n = 8).

**Figure 4:**
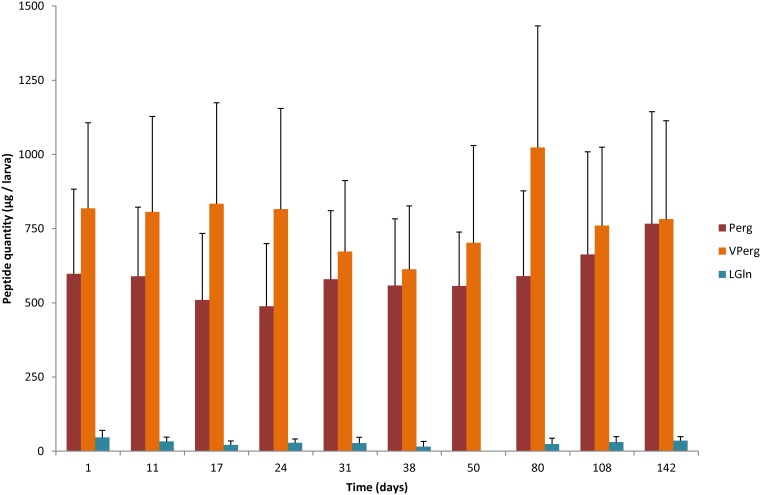
Diluted extracts from *Lophyrotoma zonalis* repeatedly analyzed to track peptide quantity over storage time. Peptide names abbreviated as in Table 1. Values (means ± SD) refer to eight extracts stored at –20°C in between the chemical analyses. Peptides shown are pergidin (Perg), 4-valinepergidin (VPerg), and lophyrotomin (LGln).

Measuring peptide stability over time revealed that concentrations of Perg, VPerg and LGln remained stable for at least one month (Fig. 4). Stability could not be assessed for dpPerg and LGlu, as these were present from the beginning at relatively low concentrations, which impeded accurate quantification.

## Discussion

In many living organisms, including sawflies, taxonomic affiliation across species is reflected in congruent chemically-based defensive strategies (*e.g.* [33]). The fact that previously only four species from two sawfly families were known to contain toxic peptides, was not representative of their actual occurrence in nature but a strong underestimation of the actual number of species containing such peptides. Our study has discovered the presence of toxic peptides in most of the analyzed species of Pergidae and Argidae (Figs 1 and 2), two families that are closely related [34]–[36]. Since the peptides were not detected in any outgroup species, it is likely that their occurrence is restricted to the two sawfly families.

The extraction procedure used here is the first that allows chemical analysis of single specimens, which offers several advantages over earlier methods described in the literature and which are all based on large amounts of oven-dried specimens. Our methodology appears as robust in that the presence or absence of at least one of the peptides was constant across all individuals of a given species. It is unlikely that a contamination of the LC-column has affected the results, since daily blanks were performed, and the samples analyzed in triplicate on different days gave, nevertheless, similar results.

The chemical analyses revealed intraspecific variation in peptide concentrations, among individuals as well as populations. The two populations of *L. interrupta* (see Appendix S1) were sampled on host plants belonging to different genera; they showed similar concentrations of VPerg but different concentrations of Perg (Fig. 1). In Australia, *L. zonalis* has the potential to poison grazing livestock, although no such case has been reported [37]. It remains unclear to what extent geographical and/or other factors may affect the chemical profiles. However, the two populations of *A. pagana*, sampled in different years and at different locations, had similar chemical profiles (Fig. 1), suggesting that there is no temporal and geographical influence on the chemistry of this species. More generally, the biosynthesis of the peptides remains unknown, not the host plant but endosymbionts being supposed to produce them [29].

Some of our results are strikingly different from those reported in the literature. Intriguingly, differences between the respective two data sources are quantitative but also qualitative. While our findings confirm that *A. pullata* contains LGln at ca. 50 µg/larva (Fig. 2 *versus* data estimated from [31]), this species was previously not reported to contain Perg, which we detected unequivocally. Other published peptide quantities are expressed in % DW, as follows: 0.01–0.07% LGln in *L. interrupta* [24], [28], 0.1% LGln in *A. pullata* [31], 0.16% LGln and 0.2% Perg in *P. flavipes* [29], and 0.36% LGlu and 0.43% of a Perg+VPerg mixture in *L. zonalis* [27]. Comparisons with our data are possible for the two *Lophyrotoma* species. In *L. interrupta* we detected VPerg and Perg but not LGln, which has been reported ever since the first publications on the chemistry of toxic peptides, and has been mentioned as the only peptide in that species [24], [25], [28]. For larvae of *L. zonalis* we obtained an FW/DW ratio of 4, so that our % FW values are equivalent to ca. 0.004% DW LGlu, 0.09% DW LGln, and 2.8% DW Perg+VPerg (see Appendix S3). Thus, there are quantitative and qualitative inconsistencies between peptide concentrations in our study and those in the literature. The differences in chemical profiles may have multiple causes and remain difficult to extricate. The methods of extracting and chemically analyzing the compounds may impact the results, and our LC-MS analyses generally seem to slightly overestimate the peptide amounts, as shown by the recovery experiments. In contrast, the ways of preparing specimens, by drying them in an oven or keeping them in ethanol, do not influence the chemical output (see Fig. 1), which corroborates a high thermostability of the peptides. The latter are chemically stable because they are water soluble but lipophilic, strongly acidic, and enzymatically non-degradable compounds [27]. Some differences between published chemical profiles and our results might also be due to misidentifications. Apart from specific taxonomic problems with individual taxa, the identification of sawfly larvae, including those in Pergidae and Argidae, still is generally hampered by the lack of suitable identification keys.

The combination of the unusual chemical properties and high toxicity of the peptides has provoked reservations against using certain pergid species as biological control agents (*e.g.*, [21], [22]). In contrast, the argid *C. janthina* has been introduced on Reunion Island to control the invasive *Rubus alceifolius* (Rosaceae) [38]. We have not seen reports of this argid affecting the local (vertebrate) fauna, although in our analyses larvae of *C. janthina* (collected from Reunion Island) contain high amounts of Perg and LGln (Fig. 2).

Considering the functioning of (natural) ecosystems, the poisoning of livestock following the ingestion of toxin-containing sawfly larvae is merely an epiphenomenon. On the scale of the larvae, the value of the toxic peptides probably lies in defense against natural enemies such as predators. The peptides were not detected in species of the pergid subfamily Perginae, but these larvae exhibit another defensive mechanism. Once disturbed, they discharge a viscous oral fluid [39], [40], perhaps as an alternative defensive strategy. In laboratory bioassays, extracts from several isolated body parts of *A. pagana* and *A. pullata* proved to be effective as feeding deterrents against ants, and the extracts also rapidly paralyzed feeding ants; both of these bioactivities are ascribed to the action of peptides [32]. The taming of aggressive behavior by ants has been documented also for oligopeptides recently isolated from frogs [6]. It is likely that predators are strongly deterred from ingesting sawfly larvae that contain toxins. In turn, this fact should keep the peptides from being disseminated widely in the food webs of natural environments.

## Conclusions

The peptides that caused the death of livestock in various regions of the world have been detected in the larvae of nearly all analyzed species of Argidae and Pergidae; this supports our initial hypothesis that the toxins occur commonly in these sawfly taxa. Concerning previous reports on such peptides in the literature, we analyzed three of the four corresponding species and found strong deviations from the published species-specific chemical profiles. Intraspecific variation, but also the methods used for chemical extractions and analyses, are possible explanations for these differing results.

## Supporting Information

Appendix S1 Table mainly containing systematic, collection, and host-plant data on the sawfly larvae used in this study.
(XLSX)

Appendix S2 Figure showing TIC chromatograms and full MS^2^ mass spectra of the five synthesized peptides.
(PDF)

Appendix S3 Table containing peptide quantities and concentrations of the five peptides in the insects analyzed by LC-MS.
(XLSX)
