# Supplementary figures and images for: Toxic Peptides Occur Frequently in Pergid and Argid Sawfly Larvae
Source: PLoS One. 2014 Aug 14;9(8):e105301. doi: 10.1371/journal.pone.0105301 (PMC4133368; doi:10.1371/journal.pone.0105301)

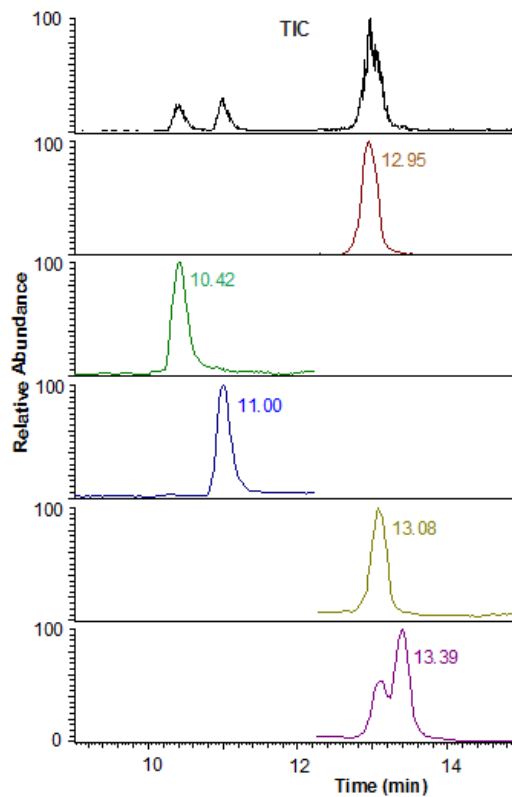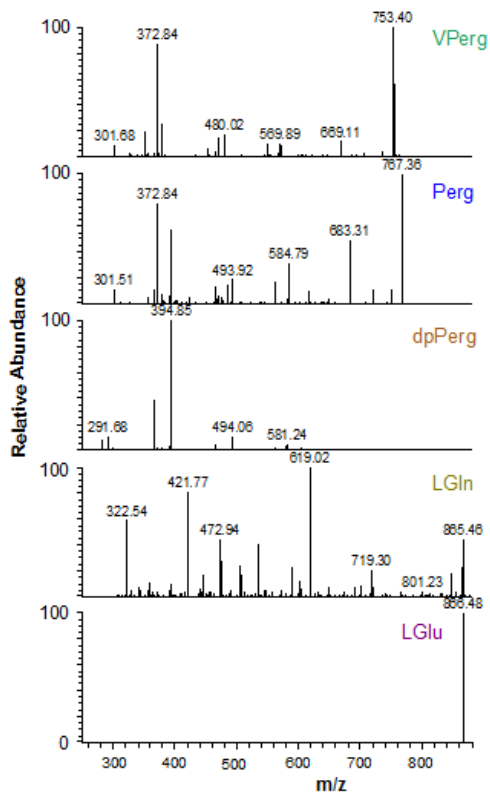

Supplement: Appendix S2 — Figure showing TIC chromatograms and full MS2 mass spectra of the five synthesized peptides. (PDF) [file pone.0105301.s002.pdf]
